# Stromal Targets for Fluorescent-Guided Oncologic Surgery

**DOI:** 10.3389/fonc.2015.00254

**Published:** 2015-11-20

**Authors:** Martin C. Boonstra, Jai Prakash, Cornelis J. H. Van De Velde, Wilma E. Mesker, Peter J. K. Kuppen, Alexander L. Vahrmeijer, Cornelis F. M. Sier

**Affiliations:** ^1^Department of Surgery, Leiden University Medical Center, Leiden, Netherlands; ^2^Department of Biomaterial Science and Technology, Targeted Therapeutics, University of Twente, Enschede, Netherlands; ^3^Antibodies for Research Applications BV, Gouda, Netherlands

**Keywords:** targeting, imaging, surgery, tumor-associated, stroma, cancer

## Abstract

Pre-operative imaging techniques are essential for tumor detection and diagnosis, but offer limited help during surgery. Recently, the applicability of imaging during oncologic surgery has been recognized, using near-infrared fluorescent dyes conjugated to targeting antibodies, peptides, or other vehicles. Image-guided oncologic surgery (IGOS) assists the surgeFon to distinguish tumor from normal tissue during operation, and can aid in recognizing vital structures. IGOS relies on an optimized combination of a dedicated fluorescent camera system and specific probes for targeting. IGOS probes for clinical use are not widely available yet, but numerous pre-clinical studies have been published and clinical trials are being established or prepared. Most of the investigated probes are based on antibodies or peptides against proteins on the membranes of malignant cells, whereas others are directed against stromal cells. Targeting stroma cells for IGOS has several advantages. Besides the high stromal content in more aggressive tumor types, the stroma is often primarily located at the periphery/invasive front of the tumor, which makes stromal targets particularly suited for imaging purposes. Moreover, because stroma up-regulation is a physiological reaction, most proteins to be targeted on these cells are “universal” and not derived from a specific genetic variation, as is the case with many upregulated proteins on malignant cancer cells.

## Background

Diagnosis, staging, and surgical planning of cancer patients increasingly rely on non-invasive pre-operative imaging techniques that provide information about tumor biology and anatomical structures (1–3). Presently, single-photon emission computed tomography (SPECT) and positron emission tomography (PET) are widely implemented imaging modalities used to provide insights into tumor location, tumor biology, and the surrounding micro-environment (1, 4). Both techniques depend on the pre-operative recognition of tumors using radioactive ligands. Various peptides and monoclonal antibodies, the latter often originally developed as therapeutic agents (e.g., cetuximab, bevacizumab, labetuzumab, rituximab, and trastuzumab), are labeled with radioactive isotopes and evaluated for pre-operative imaging purposes (4–9). However, translating information from these images to the operating theater is difficult due to alteration in body positioning, tissue manipulation by the surgeon, and the lack of sensitivity for sub-centimeter lesions. Therefore, during the actual operation, the surgeons still rely mostly on their eyes and hands to distinguish healthy from malignant tissues. In combination with the ongoing paradigm shift to more neo-adjuvant therapies like for rectal, esophageal, and breast cancer, the urge of recognition of free resection margins will become even more pronounced. An intraoperative imaging technique that can monitor tumor development in real-time will clearly contribute to the clinical establishment of “wait-and watch”-based cancer therapies (10).

In contrast to SPECT and PET, near-infrared fluorescence-guided oncologic surgery (NIRF-IGOS) is a real-time intraoperative imaging technique. NIRF-IGOS is introduced and validated in the clinic for sentinel lymph node mapping and biliary imaging and has the potential to significantly revolutionize image-guided surgery due to its key principles (11–16): first, photon absorption in living tissue is minimal between 650 and 900 nm and photon scatter is much lower in the NIRF range than in the visible spectrum. Both properties permit visualization of tumors/structures up to 5–10 mm below the surface of the tissue. Furthermore, tissue auto-fluorescence is low in the NIRF spectrum – minimizing background – and NIR light is invisible for the human eye and, therefore, does not change the surgical field, guaranteeing normal clinical workflow (17). After injection of a NIRF probe into the patient, a bright spot on a black background is detected by a camera system, which can be superimposed on the color image of the surgical field on a display (18). Combining a NIR-dye to a specific tumor-targeting ligand, like an antibody or a peptide, dramatically enhances the specificity of this technique, providing a solid real-time identification and demarcation system for the detection of tumors or nearby vital structures. This was recently shown by Rosenthal et al. who conjugated cetuximab to the NIRF dye IRDye800CW and used it to recognize surgical resection margins with sub-millimeter resolution in patients with head-and-neck cancer (19). Tumor-specific image-guided oncologic surgery (IGOS) could be considered as an extension of SPECT/PET imaging, using the same strategy regarding tumor-recognizing ligands, but applying NIR fluorophores instead of radioactivity. Compared to SPECT/PET, IGOS provides higher spatial resolution and enables direct anatomical feedback, advantageous for real-time clinical applications (2).

## Clinical Applicability

Like other novel techniques, the development and use of clinically applicable imaging systems is depending on the availability of specific anti-cancer fluorescent probes, and vice versa. Due to the increasing opportunities and indications explored, clinical fluorescence imaging systems are rapidly becoming available and the total market for image-guided surgery devices is expected to reach USD 4.8 billion in the year 2022 (www.transparencymarketresearch.com). The first open NIRF camera systems mentioned in the literature are the SPY (2003) (20), the FLARE™ imaging systems (2010) (21), Photodynamic Eye (2010) (22), and Fluobeam (2010) (23); all with their own characteristics like different wavelengths, fields of view, light sources, and working distance as extensively reviewed by Gioux et al. (24). The prices of the updated versions of these NIR fluorescence imaging systems, between $40,000 and $250,000, are relatively inexpensive when compared to clinical PET/SPECT systems. We recently validated and clinically introduced the novel ARTEMIS camera system that can be adjusted to visualize 500, 700, and 800 nm fluorophores, showing clinical feasibility for sentinel lymph node mapping and imaging of colorectal liver metastasis (25). Besides those indications, NIRF-IGOS has shown to be of advantage in breast (26), head-and-neck (19), brain, and colorectal cancer surgery (27). Moreover, there are surgical indications/approaches for which IGOS seems almost indispensable. For instance, during minimal invasive operations (laparoscopic/endoscopic), where palpation of the tissue is impossible making it difficult to recognize resection margins and small tumor nodules, and after chemo- or radiation therapies, where most of the tissue is scarred or destructed, or in cancers with prevalent inflammation, complicating the recognition of healthy and malignant tissue. Another obvious application would be in cancer types for which high numbers of positive resection margins are experienced such as with oropharyngeal or oral squamous cell carcinomas (OSCC). Of these head-and-neck cancer patients, minimal 16% have incomplete resection margins after surgery (28, 29), deteriorating patient prognosis, whereas applying broader surgical margins will result in functional impairment (30–32). Also in pancreatic cancer, surgery resections are incomplete in more than 50% of the patients, resulting in high morbidity (40–50%) and extremely low 5-year survival rates (<5%) (33).NIRF-IGOS is able to evaluate resection margins with high resolution and may result in enhanced patient survival in the near future, as incomplete resections are a strong predictor of the development of distant metastasis and subsequent decreased survival.

## Targeting of Tumor Stroma

Some tumor types over-express specific membranous proteins, like Her2/Neu for Her2 positive breast cancers and PMSA receptor or folate receptor-α as present in the majority of prostate and ovarian cancers, respectively. These targets are particularly suited for selective targeting (34–36). Unfortunately, these proteins are only present in (subsets of) those particular tumor types. This is reflected in the quest for potent targets for a broader range of tumor(s) types, resulting in an excess of pre-clinical studies published in the last years. The majority of these studies focus on “general” tumor-associated receptors, adhesion molecules, or enzymes that are located in the membrane of the majority of malignant cancer cells, such as EGFR, EpCAM, and CAIX. Examples are studies with radioactive or NIRF-labeled versions of (FDA approved) antibodies like cetuximab against EGFR and WX-G250 versus CAIX (37, 38). Because of the heterogeneity found within single tumors and between various tumor types, these studies did not generate one single omnipotent target yet. Recent alternative strategies have put effort on protein targets on non-epithelial cells, present within the tumor microenvironment.

**Figure 1:**
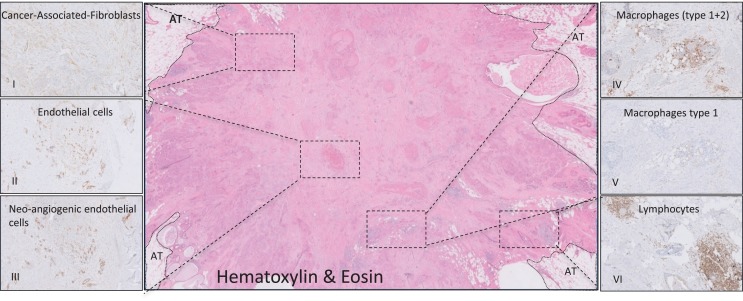
**Histological representation of targetable stromal cells**. Immunohistochemical analyses of a ductal breast carcinoma. (I, SMA) Cancer-associated fibroblasts (CAF) are disperse located through the whole tumor. The endothelial cells captured in (II, CD31) are mainly neo-angiogenic as seen in (III, CD105). (IV, DC68) Most of the macrophages present in this tumor consist of the M2 type (V, CD163). Picture (VI, CD45) shows lymphocytes that are located in conglomerates on the tumor border. AT, adipose tissue.

Our survival studies in which breast, colon, and esophageal tumors were stratified as stroma-poor (≤50%) and stroma-rich (>50%), not only indicated a strong impact of the stromal contribution to tumor behavior, but also revealed that many tumor (types) consist for a substantial part of stroma (39–42). Within these studies, the number of patients with no stroma was negligible. Further immunohistochemical analyses showed that the stroma of breast carcinomas consisted of comparable numbers of fibroblasts (including SMA-positive fibroblasts), immune cells (including type I and II macrophages) and endothelial cells (including CD105 positive endothelial cells), see Figure 1. Another tumor type eligible for stroma targeting would be pancreas cancer. The most prominent histological hallmark of pancreatic cancers is the pronounced desmoplastic reaction, consisting of fibroblasts/stellate cells, lymphatic and vascular endothelial cells, immune cells, and extracellular matrix, which could account for more than 90% of the total tumor volume (43–46).

**Figure 2:**
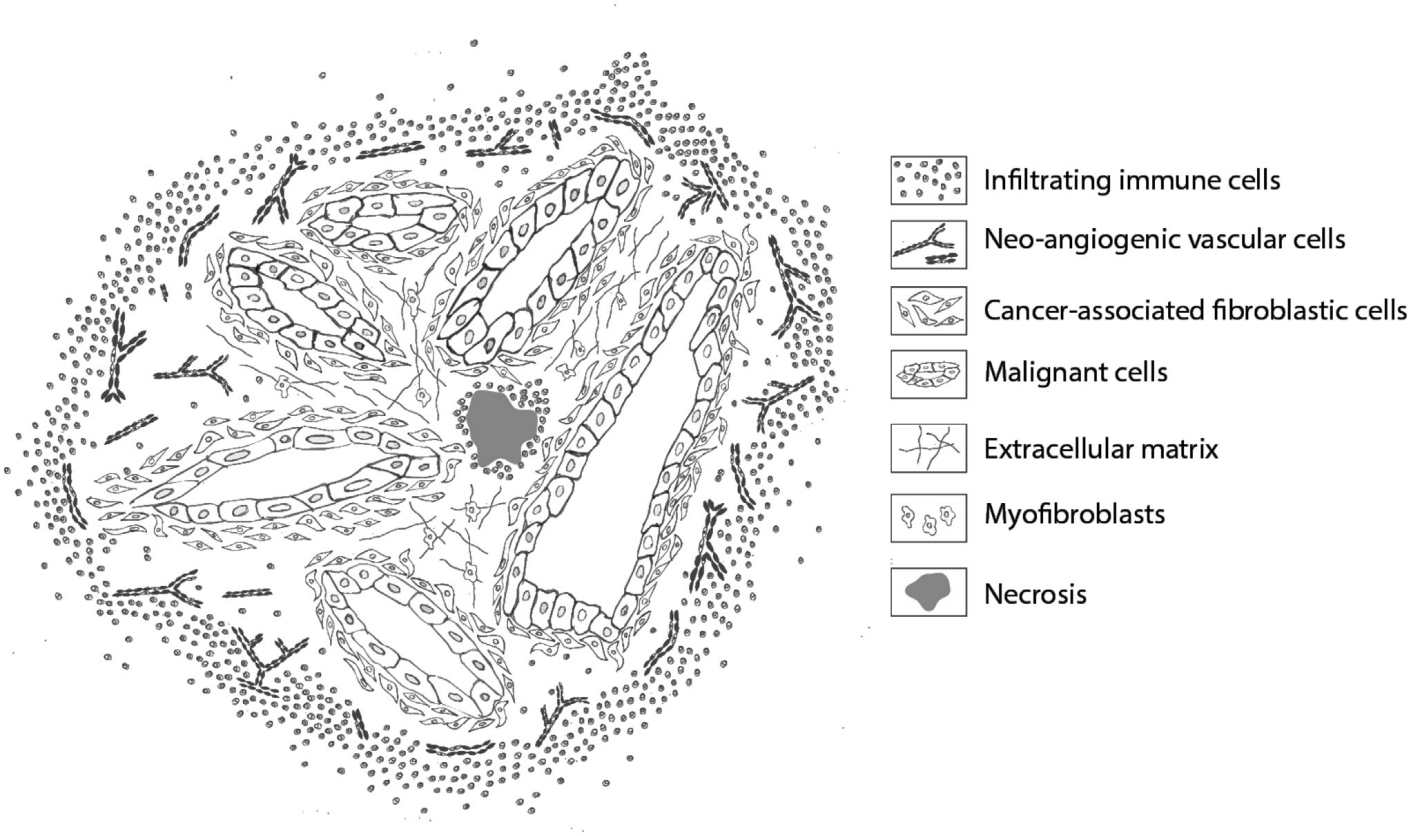
**Schematic representation of stromal cells in a ductal adenocarcinoma**. Overview of the location of the various stromal cells that can be targeted for imaging. Infiltrating immune cells are mainly located in the periphery, creating a rim around the tumor, and in necrotic areas. Neo-angiogenic endothelial cells are primarily present at the invasive front. Cancer-associated fibroblasts are mainly recognized in proximity and between malignant cells, whereas normal (myo)fibroblasts are dispersed throughout the extracellular matrix, providing rigidity and strength. Necrotic areas are mostly recognized in the tumor core.

Historically, endothelial cells have been the first tumor stromal cells to be exploited for imaging, in various types of cancer. Because of their function, mature endothelial cells are stable, inactive cells with a long lifespan. Neo-angiogenic endothelial cells, however, being tumor-induced outgrowths of existing endothelium, are activated cells with specific characteristics and protein expression and are mainly present in the periphery of the tumor (Figure 2) (47, 48). Neo-angiogenic endothelial cell associated proteins used for imaging are VEGF/VEGFR-2, α_v_β_3_ integrin and matrix metalloproteinase MMP-2 and MMP-9. The α_v_β_3_ integrin has been successfully targeted in many pre-clinical and clinical imaging studies using the peptide sequence arginine–glycine–aspartic acid (RGD), conjugated with nuclear as well as (NIR) fluorescent labels (49–54). First-in-human clinical trials are being prepared using analogs of this peptide conjugated with NIR labels. VEGF and its receptor(s) are targeted primarily via monoclonal antibodies (55). Clinical studies with anti-VEGF monoclonal antibody bevacizumab, conjugated with near-infrared IRDye800CW, are presently being performed in patients with esophageal, familial adenomatous polyposis and rectum in University Medical Center Groningen, the Netherlands (ClinicalTrials.gov). Another promising candidate target against tumor endothelium is endoglin or CD105. Pre-clinical studies in mice models have shown positive results with antibodies conjugated with PET and NIRF labels (56, 57). Dose-finding studies for therapeutic application of humanized anti-CD105 antibodies in various tumor settings have been performed, which should pave the way for the clinical usage of this target/antibody combination for tumor imaging.

Next to endothelial cells, the tumor microenvironment consists mainly of immune cells and fibroblasts, both with their own intra-tumoral distribution as schematically shown in Figure 2. Immune cells are therapeutically being targeted by CD20-directed antibodies like rituximab, ofatumumab, and obinutuzumab, especially for B cell lymphomas and leukemias. Recently, obinutuzumab has been evaluated for NIRF-based imaging of non-Hodgkin’s lymphomas (58). However, for most tumor types, CD20-based imaging is not applicable. A more suitable cell type in many tumors would be macrophages, especially tumor-associated macrophages of the M2 phenotype. M2 macrophages are not a uniform population, but several more or less common cell surface markers for this phenotype are being used for identification, i.e., CD163, CD206, and CD209. Some of these markers are being evaluated for PET and SPECT imaging, not only for oncologic purposes (59, 60). An interesting recent approach is the development of macrophage-specific NIRF dyes independent from targeting determinants (61). With respect to macrophage targeting also uPAR, the receptor for urokinase-type plasminogen activator, might be an attractive alternative. As component of the proteolytic plasminogen activation cascade, uPAR is involved in degradation/remodeling of the extracellular matrix, important for migrating cells. Therefore, uPAR is not only upregulated on invading cancer cells but also on tumor stromal cells like tumor-associated macrophages and neo-angiogenic cells (62–65). The simultaneous targeting of (invading) tumor cells as well as tumor stromal cells by a uPAR-specific probe increases the percentage of tumor mass that will be targeted and would make uPAR a pluripotent tumor target, applicable for a broad range of tumor types (66–68).

Fibroblasts, in particular, the cancer-associated fibroblasts or CAFs, are the most abundant non-immune cell type in the tumor microenvironment. CAFs are believed to develop from various origin cells and have similar characteristics as myofibroblasts (69, 70). The majority of CAFs express alpha-smooth muscle actin (α-SMA) and FSP-1, but also membrane-associated proteins are used for identification, characterization, and isolation, e.g., FAP-alpha, CXCR4/CXCL12, HGF, and PDGFR. Imaging probes based on FAP and PDGFR have shown promising results in pre-clinical studies but have not led to clinical trials yet (71–75). Considering that a number of pathways, including Hedgehog, Notch, and transforming growth factor-beta, are involved in mediating cross-talk between the malignant epithelium and its associated stroma, the list of possible stroma-derived candidate proteins for tumor imaging will expand considerably (76).

One of the major obstacles for the development and evaluation of probes for tumor stromal targets is the availability of pre-clinical validation procedures. The *in vitro* validation of the affinity/efficiency of anti-stroma cell NIRF probes can very well be established using cultures of human stromal cells. But the use of relatively simple xenograft models of human tumor cells in immunocompromised mice, as the ultimate *in vivo* proof before clinical studies, is not easily applicable for stromal cells (77). The murine stromal component formed around xenografted human cancer cells cannot be used with probes designed to target human proteins due to species specificity. On the other hand, human stromal cells are generally not suited for xenografting in mice because of the lack of human growth factors. Tumor patient-derived xenografts could provide a source of tumor stroma but are heterogeneous and black boxes with respect to which cell types are actually transplanted efficiently (78). Transplantation of human skin on mice, followed by inoculation of tumor breast cancer cells within the dermis of the transplanted skin resulted in the formation of xenografts expressing stroma and vessel elements of human origin (79). The suitability and versatility of this model was shown by imaging FAP-expressing fibroblasts using radio-labeled antibodies. Both models are physiologically acceptable, but laborious and time consuming, and probably not always necessary for imaging of specific stromal cells. These models could be simplified, however, by implanting pre-grown spheroids of human cancer cells co-cultured with tumor stromal cells in various combinations (80, 81).

## Tracer(s)/Targeting Vehicles

To design/obtain a fluorescent probe for tumor imaging, one has to make two principal decisions: which (tumor)protein will be targeted and what kind of targeting vehicle will be used to target with. As indicated above, the selection of the target seems of key importance, but also for the format of the targeting vehicle and the conjugated NIRF dye are many options available, each with their own (dis)advantages. Various types of vehicles can be used, ranging from relatively large monoclonal antibodies and antibody fragments to small peptides or RNA/DNA aptamers. Next to conventional monoclonal antibodies, a whole range of recombinant antibodies and antibody fragments are available due to advanced recombinant protein technologies. In fact, because there is no need for the Fc part of antibodies for the purpose of imaging, the antibody format has become completely dispensable, leading to a range of over 20 different non-IgG-based scaffolds such as Affimers, DARPins, and Centyrins (82, 83). These non-IgG scaffolds consist of a protein backbone with a targeting determinant that is generally selected from a library using phage, ribosome, or yeast display. Like for peptides and antibody(fragments), these scaffolds can be conjugated with fluorescent dyes, preferably from within the NIR range, for better tissue penetration and less background as discussed earlier (13). In principle, all these targeting vehicles could also be attached to nanoparticles like liposomes, dendrimers or to nano/microbubles to improve the stability and efficacy or to target more than one protein (84). The selection of a good vehicle/dye combination is complex and depends on many factors. Important characteristics are efficient tumor penetration in combination with low affinity for surrounding normal tissue and, depending on the application, a reasonable (hours) half-life in the circulation (85). The most straight forward approach, using natural ligands (or derivates), for receptors and adhesion molecules that are overexpressed in tumor cells has shown good results for, respectively, the folate receptor-α, cMet and alpha_v_beta_3_ integrin (27, 50, 86). Several therapeutic monoclonal antibodies have been investigated and show specific and sensitive tumor-binding characteristics. Because of their large size (150 kDa), antibodies possess relatively long elimination times (up to 72 h) and subsequently provide large imaging windows (24–96 h). When shorter elimination times are desired, smaller vehicles such as F(ab)s (50 kDa), scFv (27 kDa), nanobodies (27 kDa), and/or small peptides (1–2 kDa) can be addressed. In general, the use of smaller ligands increases the tumor penetration, decrease liver uptake, reduce background signals, and shorten the time between injection and imaging (87, 88). But next to size, these ligands vary considerably in physical characteristics like affinity and charge, which also have major impact on the applicability of the probe. Another important feature to consider is the possibility to conjugate the selected vehicle to fluorescent dyes. Various clinical grade (NIR) fluorophores exist that have been equipped with functional groups to enable conjugation, such as NHS-ester and maleimide reactive groups, or more recently with azide or DBCO for copper-free click chemistry. IRDye800CW is an example of a GMP-produced functionalized NIR-fluorescent fluorophore designed to have optimal *in vivo* characteristics regarding low background fluorescence, low light scattering and high signal-to-noise ratios. Alternative fluorophores, like the GMP-produced ZW800-1, show similar optical *in vivo* properties, but with different characteristics with respect to charge and polarity (89, 90).

An interesting development is the engineering of targeting vehicles that can be equipped with multiple labels. A powerful synergy can be achieved when nuclear and fluorescent imaging methods are combined, extending the pre-operative nuclear diagnostic images with real-time intraoperative imaging, leading to improved diagnosis and patient management. A number of pre-clinical studies have indicated the versatility of this concept (67, 91). Clinically, the advantages of multimodal agents have been shown in patients with melanoma and prostate cancer, but those studies used non-specific agents, following the natural lymph drainage pattern of colloidal tracers after peri-tumoral injection (92, 93).

## Conclusion

Within the next years, NIRF-based imaging will develop into one of the most valuable tools for oncologic surgeons. The evolution of the technique relies on the development of camera systems and specific targeting probes and several hurdles still have to be taken. The rapid technical developments considering LED-technology, optics, and camera systems, combined with the latest advances in image processing, warrants a prosperous contribution of the hardware. The quest for the best target(s), however, is only just begun, and also the most optimal targeting vehicle still has to be determined. The development of proper *in vivo* animal models to evaluate newly developed targeting probes seems to become the most crucial step.

## Author Contributions

MB and CS drafted the outlines of the review. MB designed the Figures and all authors contributed to the writing and approved the final version.

## Conflict of Interest Statement

The authors declare the absence of any commercial or financial relationships that could be construed as potential conflict of interest.

## Abbreviations

CAFs, cancer-associated fibroblasts; CAIX, carbonic anhydrase IX; EGFR, epidermal growth factor receptor; EpCAM, epithelial cell adhesion molecule; FAP, fibroblast activation protein; FDA, Food and Drug Administration; GMP, good manufacturing practices; IGOS, image-guided oncologic surgery; MMP, matrix metalloproteinases; NIR(F), near infrared (fluorescence); OSCC, oral squamous cell carcinomas; PDGFR, platelet-derived growth factor receptors; PET, positron emission tomography; PMSA, prostate-specific membrane antigen; RGD, arginine–glycine–aspartic acid; scFv, single-chain variable fragment (of an antibody); SMA, smooth muscle actin; SPECT, single-photon emission computed tomography; VEGF(R), vascular endothelial growth factor (receptor); uPAR, urokinase-type plasminogen activator receptor.
